# Supplementary material for: Boson-peak-like anomaly caused by transverse phonon softening in strain glass
Source: Nat Commun. 2021 Oct 1;12:5755. doi: 10.1038/s41467-021-26029-w (PMC8486772; doi:10.1038/s41467-021-26029-w)
Supplement: Supplementary file 1 — Supplementary Information [file 41467_2021_26029_MOESM1_ESM.pdf]

Supplementary Information for

Boson-peak-like anomaly caused by transverse phonon softening in strain glass

Shuai Ren,[1,+] Hong-Xiang Zong,[2,+] Xue-Fei Tao,[2] Yong-Hao Sun,[1] Bao-An Sun,[1] De-Zhen Xue,[2] Xiang-Dong Ding,[2,*] and Wei-Hua Wang[1,*]

[1] *Institute of Physics, Chinese Academy of Sciences, Beijing 100190, China*

[2] *State Key Laboratory for Mechanical Behavior of Materials, Xi'an Jiaotong University, Xi'an 710049, China*

+ These authors contributed equally to this work.

* e-mail: dingxd@mail.xjtu.edu.cn, whw@iphy.ac.cn

Supplementary Note 1: Difference of the microstructure in structural glass and strain glass

Strain glass is a glassy phenomenon in shape memory alloys, whose microstructure is crystalline, fundamentally different from the amorphous structure of structural glass. As shown in Supplementary Fig. 1, the structural glass inherits the disordering of the atomic configuration from the liquid and thereby exhibits an amorphous structure. In contrast, the strain glass inherits the crystalline structure of the parent phase[1, 5]. Therefore, the average structure of strain glass is a simple crystalline structure, and the frozen martensitic nanodomains are the nanosized distorted regions with local strain order, which are coherent with the parent phase matrix.

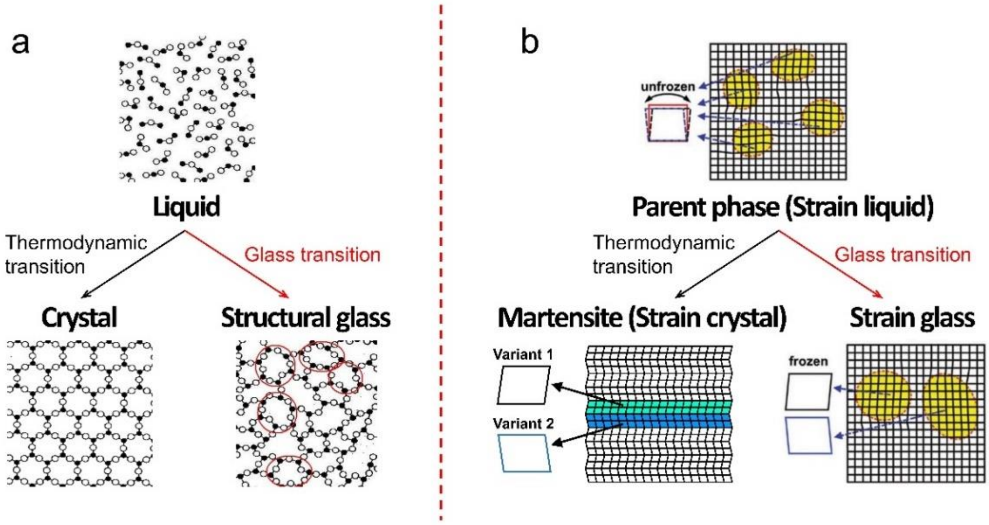

Supplementary Figure 1. Schematic pictures of microstructure in (a) structural glass and (b) strain glass. The microstructure of structural glass in (a) is amorphous, whereas the microstructure of strain glass in (b) is crystalline.

Supplementary Note 2: Phase diagram of $Ti_{50-x}Ni_{50+x}$ shape memory alloy

Supplementary Figure 2a exhibits the phase diagram of $Ti_{50-x}Ni_{50+x}$ shape memory alloys, in which the strain glass appears when x exceeds a critical concentration around x = 1.5, consistent with the previous work[31]. Supplementary Figure 2b exhibits the enthalpy change (ΔH) as a function of x, which is calculated by the heat flow curves in the inset. The ΔH gradually decreases to zero as x increases from 0 to 1.5, indicating that the martensitic transformation is gradually suppressed with the increase in x.

Supplementary Figure 2c exhibits the frequency-dependent behavior of the storage modulus and tan δ: the dip temperatures (or peak temperatures) of the storage modulus (or tan δ) curves decrease when the frequency decreases. This is a typical evidence of strain glass, as reported by previous studies[1, 4, 5]. The frequency-dependent behavior in strain glass indicates that the strain glass transition is a freezing transition from the parent phase which can be considered as a "strain liquid" phase[1], so the slowing-down of dynamics during the strain glass transition resembles the primary α-relaxation in metallic glass. By fitting the Vogel–Fulcher relation ($\omega = \omega_0\exp[-E_a/k_B(T_g-T_0)]$), the ideal freezing temperature T_0 is determined to be ~172 K, consistent with the value in the previous studies[31].

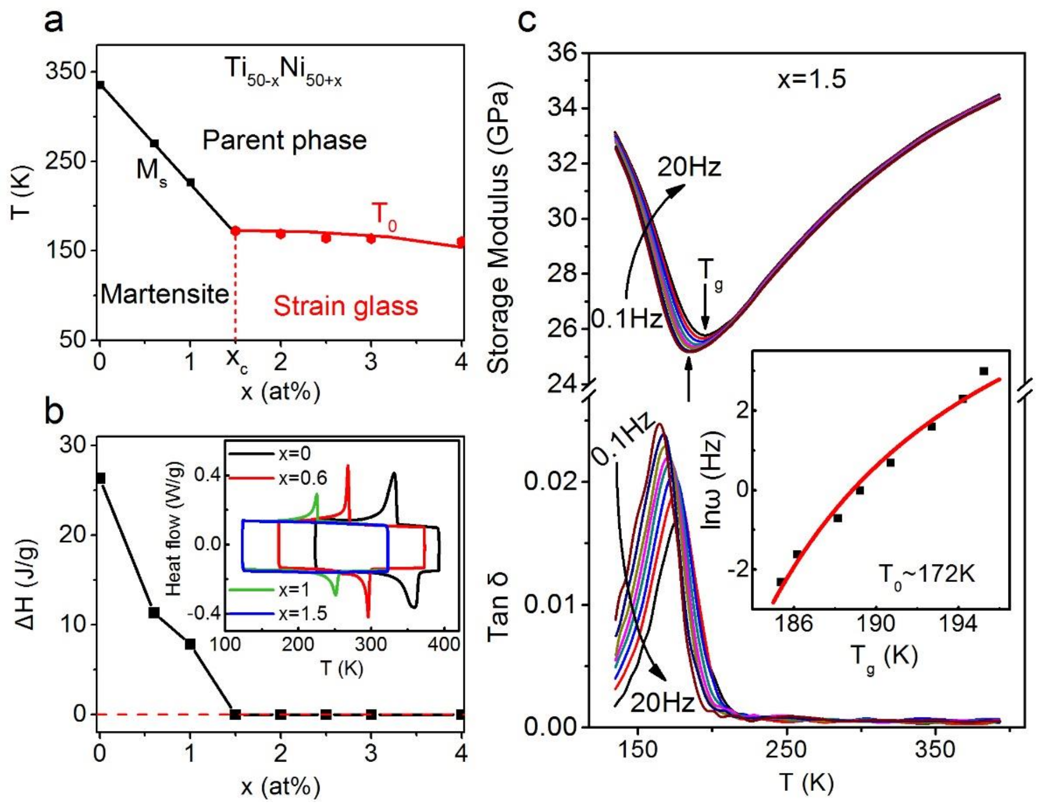

Supplementary Figure 2. Phase diagram of $Ti_{50-x}Ni_{50+x}$ strain glass alloys and supporting data. a. The phase diagram of $Ti_{50-x}Ni_{50+x}$ alloys. The crossover between the martensite and strain glass is around $x = 1.5$. **b.** The enthalpy change (ΔH) as a function of x, which corresponds to the area under the heat flow peaks in the inset. **c.** The frequency dependence of the storage modulus and tan δ at $x = 1.5$. The inset shows the fitting of Vogel–Fulcher relation of frequency as a function of T_g. The ideal freezing temperature T_0 is around 172 K.

Supplementary Note 3: The boson peak in $Zr_{50}Cu_{40}Al_{10}$ bulk metallic glass (BMG)

The $Zr_{50}Cu_{40}Al_{10}$ BMG is determined by the DSC curve and XRD pattern in Supplementary Fig. 3a. Supplementary Figure 3b exhibits the linear fitting of C_p/T vs. T^2 for both the BMG and its corresponding crystal. The reduced specific heat $(C_p\text{-}\gamma T)/T^3$ as a function of T is shown in Supplementary Fig. 3c. The boson peak of the $Zr_{50}Cu_{40}Al_{10}$ BMG is also manifested as a broad hump, lending support to the BP-like anomaly in strain glass.

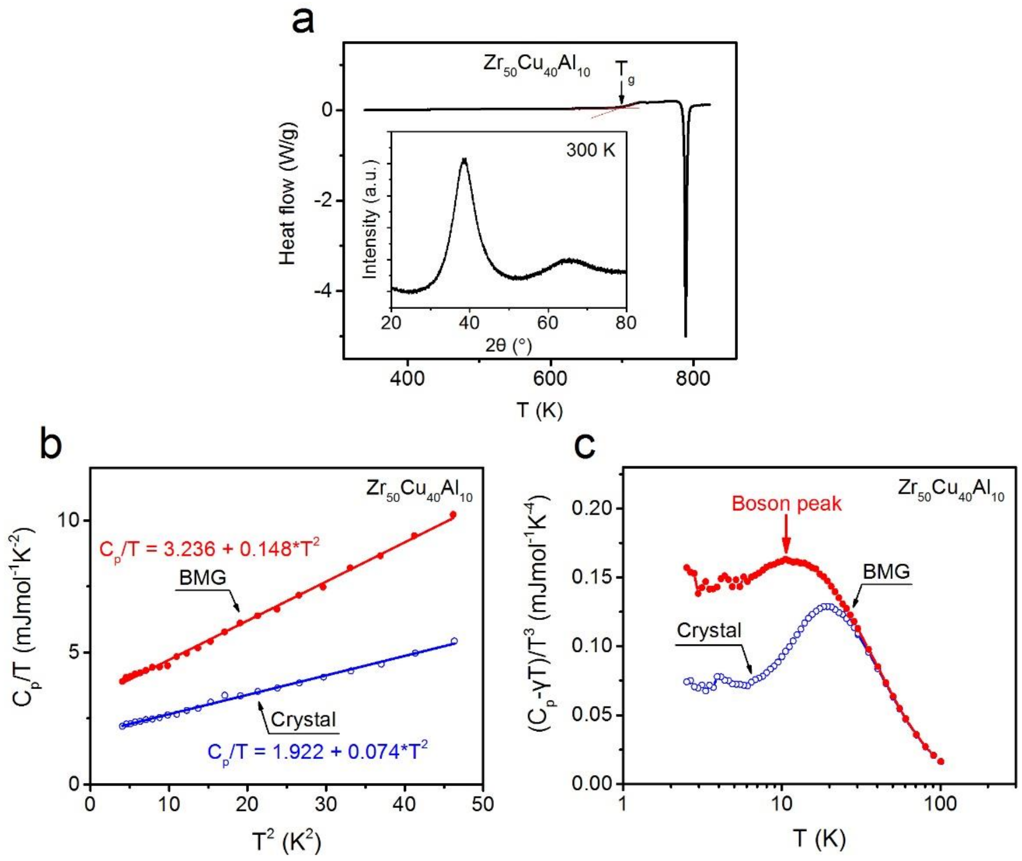

Supplementary Figure 3. Boson peak of the $Zr_{50}Cu_{40}Al_{10}$ BMG. a. DSC curve as well as the XRD pattern of the $Zr_{50}Cu_{40}Al_{10}$ BMG. **b.** C_p/T vs. T^2 relation with the C_p data below 7 K. The linear fitting follows the relation $C_p/T=\gamma+\beta T^2$. **c.** The plot of $(C_p\text{-}\gamma T)/T^3$ vs. T for the $Zr_{50}Cu_{40}Al_{10}$ BMG and its corresponding crystal. A broad hump, i.e., the boson peak, takes place around 10 K for the $Zr_{50}Cu_{40}Al_{10}$ BMG.

Supplementary Note 4: BP-like anomaly in another $Ti_{50}Pd_{50-x}Cr_x$ strain glass alloys

According to Ref. 46, the critical level of Cr separating the martensitic phase and

strain glass in $Ti_{50}Pd_{50-x}Cr_x$ alloys is around $x = 9$. In this strain glass system, an obvious hump is also observed around 10 K in strain glass, which indicates that the BP-like anomaly is a universal behavior in strain glasses, and does not rely on any specific system.

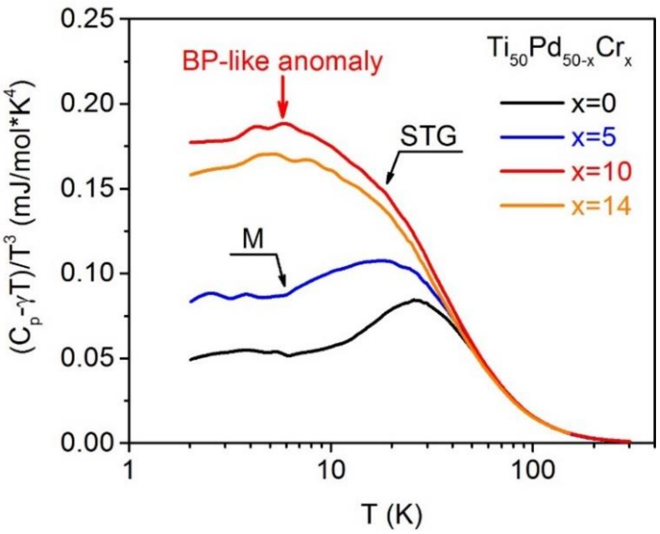

Supplementary Figure 4. Reduced specific heat $(C_p-\gamma T)/T^3$ as a function of T in $Ti_{50}Pd_{50-x}Cr_x$ alloys. A BP-like anomaly is observed in the strain glass compositions ($x = 10$, 14).

Supplementary Note 5: Calculated Phase diagram of the $Zr_{100-x}Ni_x$ model system

According to Ref. 36, upon the increase of Ni concentration, the low temperature phase changes from the martensitic phase to strain glass with a critical defect concentration of 4.0%.

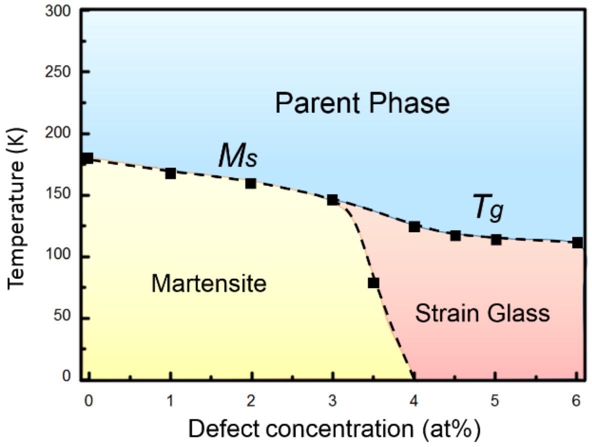

Supplementary Figure 5. Calculated phase diagram of $Zr_{100-x}Ni_x$ model alloys.

Supplementary Note 6: Transverse dynamical structure factors $S_T(k, \omega)$ of the

[110] direction

The curves of the transverse dynamical structure factors $S_T(k, \omega)$ of the [110] direction actually exhibit two peaks. The lower peak corresponds to the basal mode with displacements matching the martensitic transformation (the TA_2 mode), while the higher peak corresponds to the other TA branch along [110], namely the TA_1 mode. For the sake of clarity, only the $S_T(k, \omega)$ of the TA_2 mode is presented in Fig. 5a.

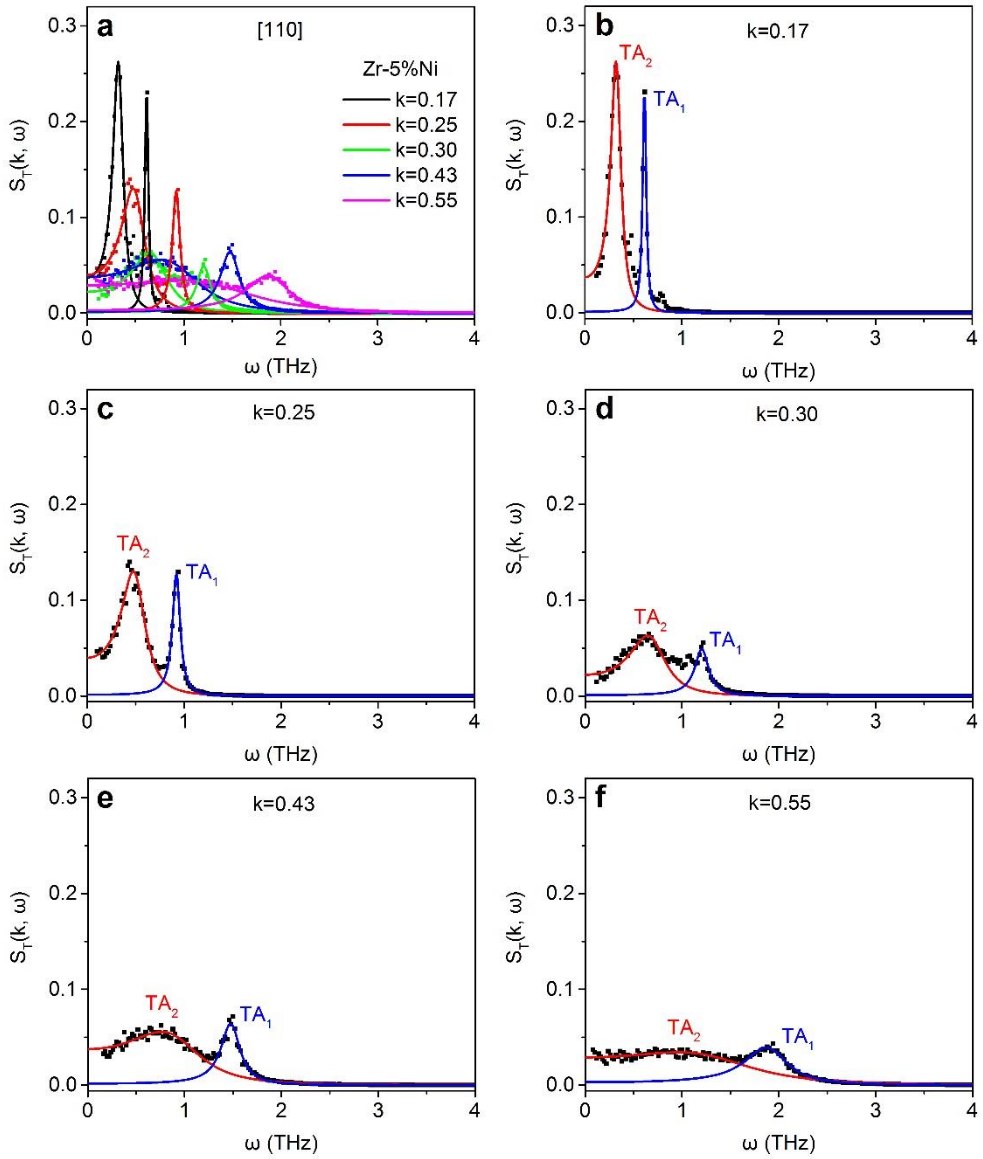

Supplementary Figure 6. Transverse dynamical structure factors $S_T(k, \omega)$ of the [110] direction. a. The complete $S_T(k, \omega)$ curves for several *k* values. **b-f.** The $S_T(k, \omega)$ curves for (**b**) k=0.17, (**c**) k=0.25, (**d**) k=0.3, (**e**) k=0.43, and (**f**) k=0.55, respectively.

Reference

46. Zhou, Y. M. *et al.* High temperature strain glass in $Ti_{50}(Pd_{50-x}Cr_x)$ alloy and the associated shape memory effect and superelasticity. *Appl. Phys. Lett.* **95**, 151906 (2009).
