# Supplementary figures and images for: Boson-peak-like anomaly caused by transverse phonon softening in strain glass
Source: Nat Commun. 2021 Oct 1;12:5755. doi: 10.1038/s41467-021-26029-w (PMC8486772; doi:10.1038/s41467-021-26029-w)

## Slide 1
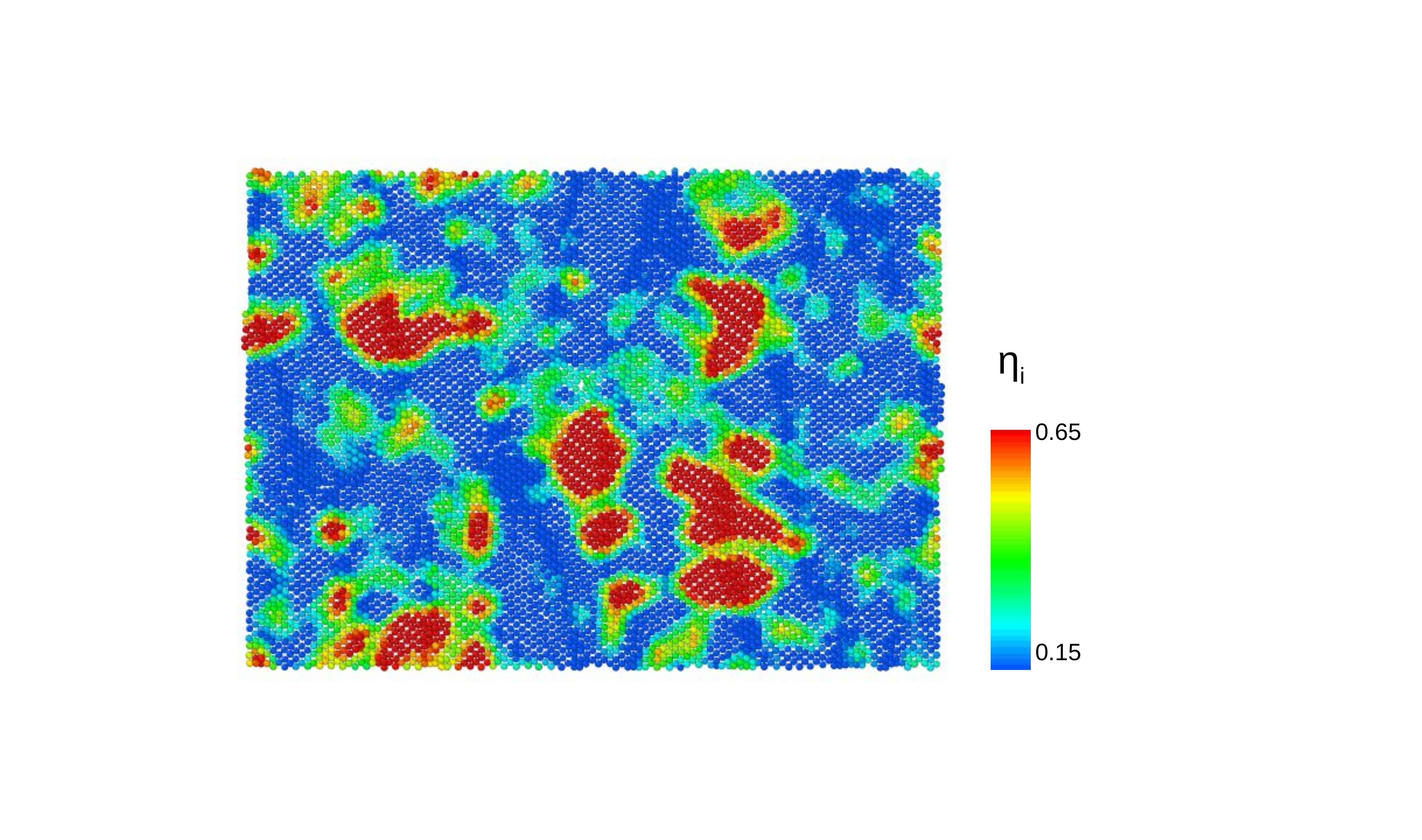

0.65
0.15
ηi

## Slide 2
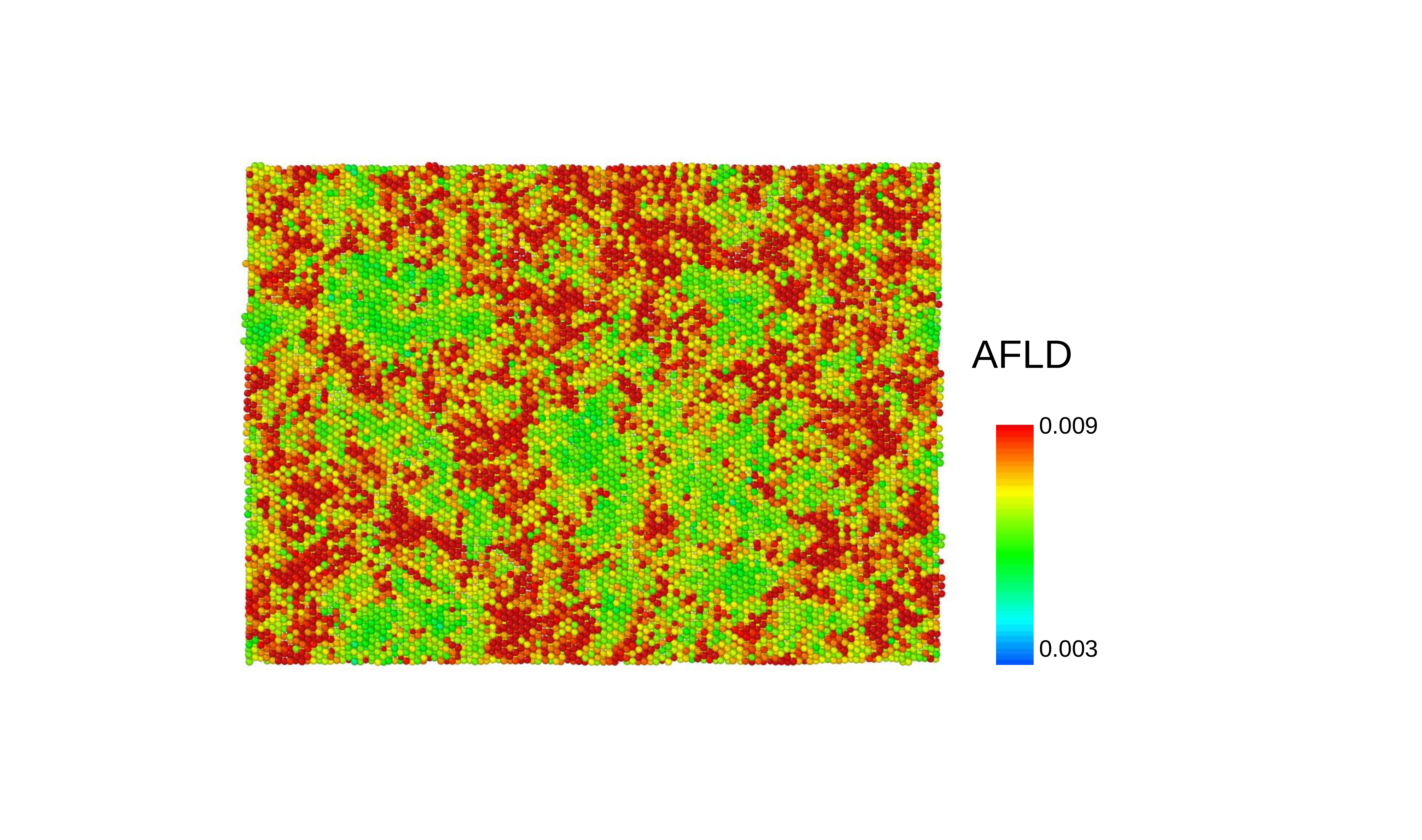

AFLD
0.009
0.003

Supplement: Supplementary file 3 — Source Data [file 41467_2021_26029_MOESM3_ESM.zip › Figures-data/Figure 4c and 4d.pptx]
